# Mechanical circulatory support in cardiogenic shock: microaxial flow pumps for all and VA-ECMO consigned to the museum?

**DOI:** 10.1186/s13054-024-04988-y

**Published:** 2024-06-20

**Authors:** Daniel De Backer, Dirk W. Donker, Alain Combes, Alexandre Mebazaa, Jacob E. Moller, Jean-Louis Vincent

**Affiliations:** 1 Department of Intensive Care, CHIREC Hospitals, Université Libre de Bruxelles, Boulevard du Triomphe 201, 1160 Brussels, Belgium; 2 Intensive Care Center, University Medical Center Utrecht, Utrecht, The Netherlands; 3 Cardiovascular and Respiratory Physiology, TechMed Center, University of Twente, Enschede, The Netherlands; 4 Sorbonne Université, INSERM Unité Mixte de Recherche (UMRS) 1166, Institute of Cardiometabolism and Nutrition and Service de Médecine Intensive-Réanimation, Hôpital Pitié-Salpêtrière, Sorbonne Université Assistance Publique-Hôpitaux de Paris, Sorbonne Université, INSERM, Paris, France; 5 Université Paris Cité, Inserm 942 MASCOT, Hôpitaux Universitaires Saint-Louis and Lariboisière, Paris, France; 6 Heart Center, Department of Cardiology, Copenhagen University Hospital Rigshospitalet, Copenhagen, Denmark; 7 Department of Cardiology, Odense University Hospital, Odense, Denmark; 8 Department of Intensive Care, Erasme University Hospital, Université Libre de Bruxelles, Brussels, Belgium

## Introduction

Mortality rates in cardiogenic shock remain high, especially in patients with SCAI shock stages C to E [1]. When hemodynamic status does not improve or worsens despite optimal fluid, inotrope and vasopressor administration, mechanical circulatory support (MCS), most commonly with veno-arterial extracorporeal membrane oxygenation (VA-ECMO) or a microaxial flow pump (MFP), is often used as rescue therapy. Although several observational studies have suggested survival benefits with VA-ECMO use in cardiogenic shock [2, 3], these effects have not been confirmed in randomized controlled trials (RCTs) [4–7]. In a metaanalysis aggregating individual patient data from 567 patients with acute myocardial infarction related cardiogenic shock (AMICS) from 4 RCTs, there was no significant reduction in 30-day mortality with early use of VA-ECMO (OR 0.93; 95% CI 0.66–1.29) [8]. However, in a recent RCT comparing MFP use to usual care in 355 patients with AMICS (DanGer Shock [9]), MFP-treated patients had lower 180-day all-cause mortality (45.8% versus 58.5%; hazard ratio, 0.74; 95% CI 0.55–0.99; *P* = 0.04). Does this imply that VA-ECMO should be abandoned [10] and MFPs used for MCS in all patients with AMICS? We are not sure.

### Scrutinizing the RCT evidence

There are numerous caveats with the recent RCTs on MCS in cardiogenic shock. First, they did not compare early use of MCS versus medical therapy alone, but rather MCS versus “medical therapy assisted by MCS at the physician's discretion”. Indeed rescue VA-ECMO was used in 39% of control patients in one study [4], and rescue MCS (26 VA-ECMO and 28 MFP) was applied in 26% of control patients in another [5]. In the recent DanGer Shock trial [9], VA-ECMO was used in 19% of control patients and a different MFP in 5% (Table 1).

**Table 1:** Main differences in the three largest randomized controlled trials (RCTs) on mechanical circulatory support (MCS) in cardiogenic shock

	Ostadal et al. [4]	Thiele et al. [5]	Moller et al. [9]
MCS type	VA-ECMO	VA-ECMO	MFP
Patients	SCAI D-E	SCAI C-E (SCAI C 53%)	SCAI C-E (SCAI C 55%)
Cardiac arrest exclusions (proportion of included patients who were post-CA)	Comatose after cardiac arrest excluded (post-CA 11%)	CPR > 45 min excluded (post-CA 78%)	Comatose after cardiac arrest excluded (post-CA 20%)
Mechanical ventilation at inclusion	70%	88%	18%
Unloading strategy	22%	6%	Not relevant
Rescue MCS in control group	Rescue VA-ECMO 39%	Rescue VA-ECMO 13% Rescue MFP 13%	Rescue VA-ECMO 13%
Additional MCS in intervention group	0%	0%	Rescue VA-ECMO 12% Other MFP 16%

A second caveat is whether early systematic introduction of MCS reflects actual clinical practice. In RCTs, MCS use is applied per protocol as soon as the inclusion criteria are met. In the large ECLS-SHOCK trial [5], VA-ECMO was indicated in the presence of low blood pressure with or without vasopressors (no minimal dose mentioned), blood lactate levels > 3 mmol/L, and signs of altered organ perfusion. Cardiac output measurements (or echocardiographic surrogates such as left ventricular outflow tract velocity time integral-VTI) were not required, yet an impaired left ventricular (LV) ejection fraction may be associated with preserved cardiac output. Furthermore, systolic blood pressure was > 120 mmHg in 25% of the patients who received VA-ECMO with a median of around 100 mmHg at randomization. Many of these patients may therefore have had relatively preserved stroke volume and adequate tissue perfusion despite persistent high lactate levels (which may take time to normalize), suggesting a good response to initial therapy and raising questions about whether they really needed MCS. Additionally, the RCT design does not allow a “personalized” approach to MCS selection. In the intervention arm, patients immediately receive one type of MCS as soon as they meet the entry criteria. In real-life clinical practice, physicians choose between different MCS strategies according to patient characteristics (e.g., MFP in patients with LV dilation or severe mitral regurgitation, VA-ECMO in conditions of biventricular dysfunction or associated hypoxemia) [11].

One may therefore question whether the RCT design, with per-protocol use of a single type of MCS in all patients in the intervention group and on demand use of “rescue MCS” in the control group, is optimal to assess the utility of MCS in shock.

### Could other study designs provide better answers?

Adaptive platform trials may be an alternative to take into account patient heterogeneity and optimal MCS selection. Other initiatives that challenge traditional methodologies are being developed, including synthetic data and virtual trials, computational physiological models, and digital twin/shadow approaches. Unfortunately, in many non-randomized designs, adjustments for confounders are often incomplete with resultant risk of bias.

### Will aggregating current data help much?

Aggregating trial data in individual patient metaanalyses [8] may enable the overall effects of the intervention to be collated, overcoming some of the limitations of the individual trials, including limited power from small sample sizes, and identifying signals undetected in the separate studies. However, the available studies are highly heterogeneous and some imbalances in factors influencing outcomes may thus remain (Table 1). Indeed, inclusion criteria varied, with one study including patients with SCAI stages D-E [4] and another SCAI C-E [5]. Similarly, comatose survivors after cardiac arrest were not included in one study [4] but were in another [5], in which cardiac arrest had occurred in 78% of the included patients. ECMO management also varied, with LV venting performed in 22% of patients in one trial [4] and in 6% in another [5]. Similar concerns apply to network meta-analyses [12]. Moreover, disease severity is rarely reported or adjusted for but can impact mortality and hence influence the results. For example, an ENCOURAGE score < 10 prior to ECMO implantation was associated with mortality < 5%, whereas a score ≥ 28 was associated with mortality of 80% [13]. Finally, selection bias may also have occurred in some trials, especially those stopped prematurely because of low inclusion rates.

Bayesian analysis of individual studies or Bayesian meta-analysis may be helpful to better inform the likelihood of benefit, as has been performed with other types of extracorporeal support [14], but will not overcome the intrinsic limitations of the studies.

### Should VA-ECMO be consigned to the museum?

VA-EVMO was used as a rescue strategy in many MCS trials, so it is difficult to determine what the mortality of the control group would have been without ECMO. Furthermore, in the DanGer Shock trial, VA-ECMO was used in 12% of the patients allocated to the intervention arm, suggesting that in one in seven patients, VA-ECMO had to be added because the MFP did not provide adequate tissue perfusion.

Moreover, in a recent survey, only a small proportion (~ 20%) of cardiogenic shock episodes were AMI-associated [15], and many of these patients will also have experienced cardiac arrest. Patients with other etiologies of cardiogenic shock and patients not fully awake after cardiac arrest were not included in the DanGer Shock study [9]. Most patients with cardiogenic shock after resuscitated cardiac arrest who have uncertain neurologic function and patients with refractory cardiac arrest are currently treated with VA-ECMO as first-choice MCS.

A real concern is how the results of such trials will be interpreted by regulatory bodies, healthcare insurances providers, or lawyers. If it is considered, based on the existing data, that VA-ECMO does not improve survival and may be associated with risks, the indication for VA-ECMO in AMICS, or other types of cardiogenic shock, may be restricted or even prohibited in the future, depriving many patients from potentially lifesaving procedures in emergency situations outside the RCT setting.

### Toward “personalized” management: selecting the most appropriate MCS for a patient in cardiogenic shock

The typical indication for MCS is cardiogenic shock not responding (SCAI stage D and E) or responding insufficiently (some of SCAI stage C) to adequate medical therapy. These patients usually have low stroke volume (≤ 30 mL) reflected by a low LV velocity time integral (VTI < 10 cm).

**Fig. 1:**
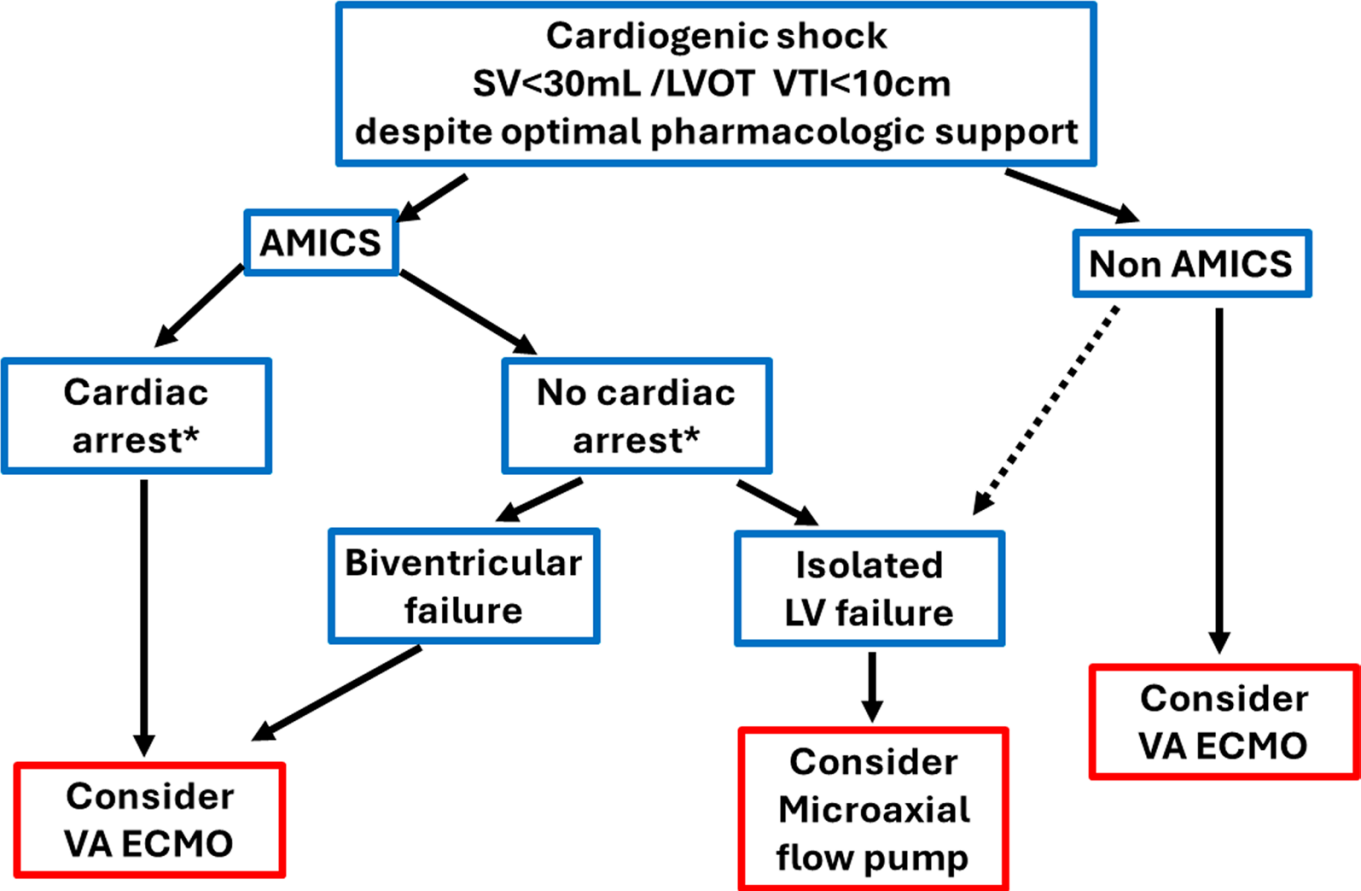
Suggested use of veno-arterial extracorporeal membrane oxygenation (VA-ECMO) and microaxial flow pumps (MFPs) in cardiogenic shock. Patients with cardiogenic shock not responding to adequate therapy may be considered for mechanical circulatory support (MCS). Non-response to adequate therapy is suggested by persistent low stroke volume (left ventricular outflow tract velocity time interval [LVOT VTI) associated with signs of tissue hypoperfusion despite optimal administration of inotropes and vasopressors. The suggested cut-offs are illustrative and should not be considered as hard cut-offs. Some alternative combinations of hemodynamic factors may also be considered. Patients with significant valvular disease or tamponade are excluded from this diagram. *Patients awake after short episode of cardiac arrest may be considered as patients without cardiac arrest. SV stroke volume; AMICS acute myocardial associated cardiogenic shock; LV left ventricle

Selection of the type of MCS should ideally be based on the mechanism underlying the shock (predominant LV dysfunction vs biventricular or predominant right ventricular dysfunction, ongoing resuscitation or prolonged cardiac arrest, hypoxemia, severity of organ dysfunction, comorbidity, …) (Fig. 1). Addition of a second type of MCS may sometimes be justified, for example for unloading during VA-ECMO or for right ventricular dysfunction/insufficient flow/hypoxemia during MFP support.

## Conclusion

Current evidence does not support the systematic use of VA-ECMO in AMICS, but it remains clinically useful when optimal medical therapies fail. It is likely that the real benefit of VA-ECMO is difficult to show in RCTs, especially as these devices will likely continue to be used as bailout strategies. Currently, both VA-ECMO and MFP have a role to play in the therapy of severe cardiogenic shock, and in daily practice we should therefore optimize how and which MCS is selected, as well as how MCS patients are managed to limit the high rate of complications.

## Data Availability

Not applicable.
